# Developing novel non-assistant help operation in dual-portal robotic-assisted thoracic surgery (neoDRATS)

**DOI:** 10.1016/j.xjtc.2024.07.019

**Published:** 2024-08-05

**Authors:** Hideki Ujiie, Hiroki Ebana, Jun Suzuki, Masato Chiba, Hikaru Watanabe, Aki Kobayashi, Satoshi Shiono, Yasuhiro Tsutani, Tatsuya Kato

**Affiliations:** a Department of Thoracic Surgery, Hokkaido University Hospital, Sapporo, Japan; b Department of Thoracic Surgery, Tokyo Metropolitan Bokutoh Hospital, Tokyo, Japan; c Department of Surgery II, Faculty of Medicine, Yamagata University, Yamagata, Japan; d Division of Thoracic Surgery, Department of Surgery, Kindai University, Osaka, Japan

**Keywords:** robotic-assisted thoracic surgery, uniportal robotic-assisted thoracic surgery, dual-portal robotic-assisted thoracic surgery, uniportal video-assisted thoracic surgery

## Abstract

**Objective:**

To introduce and evaluate the non-assistant help operation in dual-portal robotic-assisted thoracic surgery (neoDRATS), a novel technique designed to eliminate the need for skilled assistants by using all 4 robotic arms independently during anatomical lung surgery.

**Methods:**

Patients were placed in the lateral decubitus position under general anesthesia with single-lung ventilation. The da Vinci Xi Surgical System was used, with specific configurations for right- and left-side operations. The neoDRATS technique used a 4-cm working port and a 1.8-cm secondary port, with detailed guidelines for optimal setup and robotic arm manipulation.

**Results:**

The neoDRATS approach demonstrated successful surgical outcomes without the need for a skilled assistant. The use of a 0° camera and careful placement of instruments minimized interference within the thoracic cavity. The technique provided smooth operability and minimized postoperative discomfort. Video demonstrations of right and left upper lobectomies are provided to illustrate the approach.

**Conclusions:**

NeoDRATS offers a practical, safe, and minimally invasive alternative to conventional multiportal and uniportal robotic-assisted thoracic surgeries. This technique simplifies the surgical process, particularly in settings with limited availability of skilled assistants, and represents a significant advancement in robotic thoracic surgery. Further refinement and clinical integration of neoDRATS are anticipated as robotic innovations continue to evolve.

**Figure:**
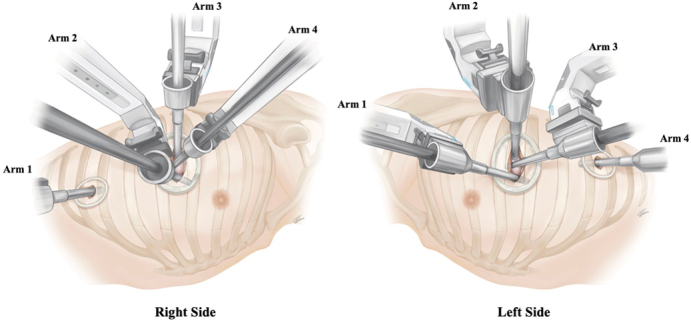
Robotic arm placement of neoDRATS (*right* and *left side*).

**Central Message**

The non-assistant help operation in dual-portal robotic-assisted thoracic surgery (neoDRATS) appears to eliminate the need for skilled assistants and seems easier to perform compared with URATS.

**Perspective**

From the perspective of operational efficiency and resource management, the non-assistant help operation in dual-portal robotic-assisted thoracic surgery (neoDRATS) is advantageous as it potentially reduces the dependency on skilled assistants and simplifies the procedure in comparison with uniportal RATS.

In recent years, there has been increasing interest in robotic-assisted thoracic surgery, particularly using the conventional multiportal method. However, the development of uniportal robotic-assisted thoracic surgery (URATS), which uses a single incision and offers better emergency-conversion capabilities, marks a significant step toward less-invasive procedures.^1^^,^^2^

Despite its benefits, URATS presents specific challenges, including mastering a steep learning curve, requiring skilled assistants, and managing instrument collisions. These factors impede its broader adoption owing to the associated high costs and the demand for specialized training. Moreover, leveraging our expertise in uniportal video-assisted thoracic surgery (UVATS) and traditional robotic techniques, we shifted to a biportal approach for RATS, now termed dual-portal robotic-assisted thoracic surgery (DRATS).^3^ This method incorporates an extra port to reduce the risks associated with the use of robotic staplers and potential interference between robotic arms. Compared with URATS, instrument collisions and interfacing between robotic arms can be reduced because the robotic stapler is inserted from the added port. This method has been validated as safe and effective for anatomical lung resections and has demonstrated excellent perioperative outcomes in multicenter studies conducted in Japan. Nonetheless, DRATS also poses challenges, including the necessity for assistants with expertise in UVATS.

To overcome these obstacles, we propose a novel technique, termed non-assistant help operation in dual-portal robotic-assisted thoracic surgery (neoDRATS). This technique uses all 4 robotic arms to create surgical fields and perform anatomic lung surgery independently, eliminating the need for a skilled assistant. Furthermore, it uses the same incisions as those in DRATS. This article describes the neoDRATS surgical technique.

## Surgical Preparation and Technique

This study was conducted in accordance with the Declaration of Helsinki (revised in 2013). This study was approved by the institutional review board of the Ethics Committee of Hokkaido University Hospital (no. 023-0172, August 29, 2023) and Tokyo Metropolitan Bokutoh Hospital (no. 05-030) on behalf of the participating institutions, and individual consent was waived for this retrospective analysis. Informed consent for surgery was obtained from all the patients. On the basis of our experience, we suggest the following guidelines for establishing an optimal setup for neoDRATS.

**Figure 1:**
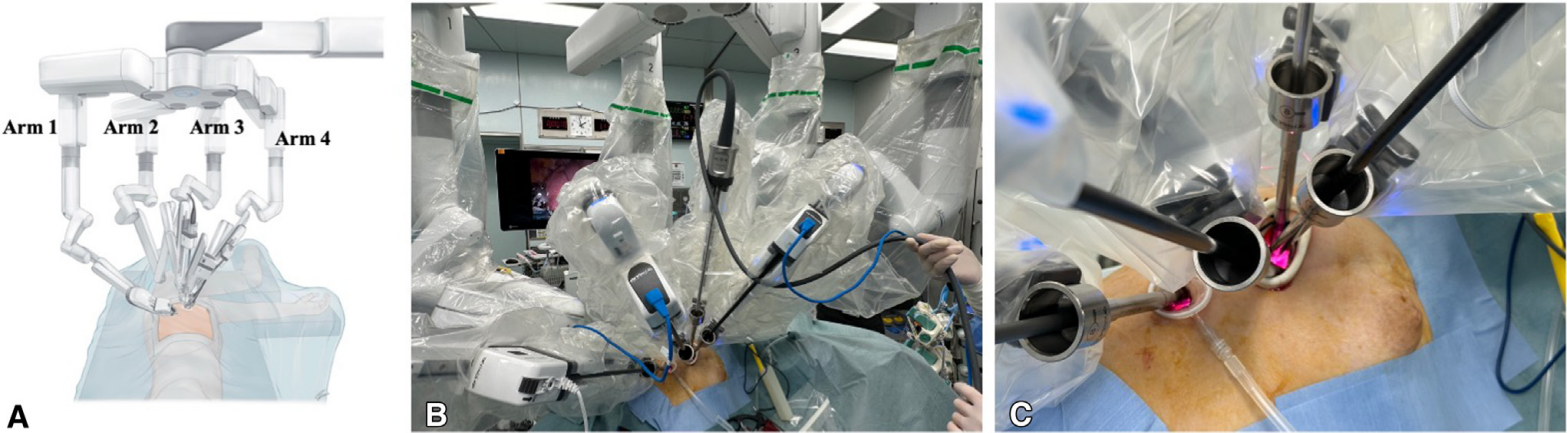
Right-side configuration. A, Illustration showing the port placement and setup of robotic arms on the right side. Arm 1 is used for the left hand, arm 2 is used for the right hand, arm 3 is for the camera, and arm 4 is for the retraction. B, The camera is normally placed in the posterior part of the incision to allow the other robotic instruments working below the camera in the same incision to be parallel along the sagittal plane. C, Enlarged view of the port placement. Arm 4 is used for the retraction as is the role of the assistant in original dual-portal RATS. This retraction arm is very helpful in obtaining better exposure. Arm 1 and arm 2 robotic instruments are used through another incision that opens wide toward the ventral side to prevent interference.

**Figure 2:**
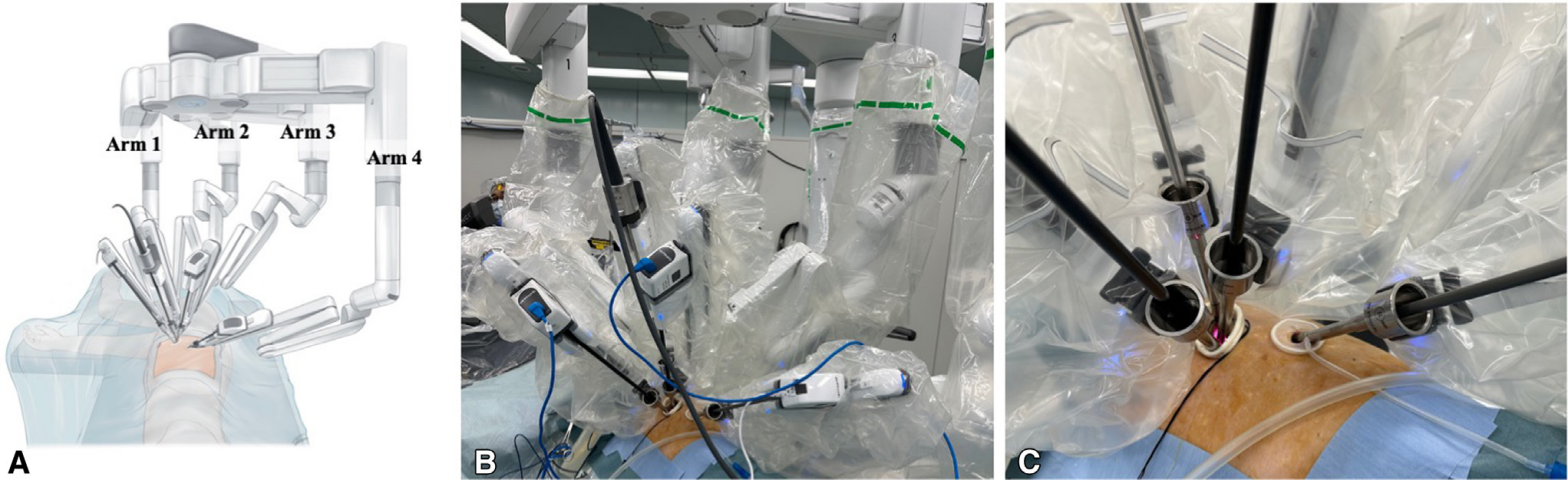
Left-side configuration. A, Illustration showing the port placement and setup of robotic arms on the left side. Arm 1 is used for the left hand, arm 2 is used for the camera, arm 3 is for the retraction, and arm 4 is for the right hand. B, The camera is normally placed in the posterior part of the incision to allow the other robotic instruments working below the camera in the same incision to be parallel along the sagittal plane. C, Enlarged view of the port placement. Arm 3 is used for the retraction as is the role of the assistant in original dual-portal RATS. This retraction arm is very helpful in obtaining better exposure. Arm 1 and arm 4 robotic instruments are used through another incision that opens wide toward the ventral side to prevent interference.

Figure 1, *A*, and Figure 2, *A*, show typical images of the body surface during neoDRATS on the right and left sides, respectively. The patients were placed in the lateral decubitus position under general anesthesia with single-lung ventilation. The da Vinci Xi Surgical System (Intuitive Inc) was positioned posterior to the patient, depending on the size of the operative room and location of the system in each hospital, with the boom of the patient cart rotated 90° toward the patient's head. Unlike standard RATS procedures, conventional targeting and carbon dioxide insufflation were omitted; instead, the laser crosshair was aligned with the upper portion of the posterior skin incision, running parallel to the intercostal space.

In the neoDRATS approach for lobectomy and segmentectomy procedures, an Alexis wound retractor XS (Applied Medical) was used to establish a working port measuring 4 cm in the fifth intercostal space along the middle axillary line for all lung lobes and segments. A secondary port measuring 1.8 cm was placed in the seventh or sixth intercostal space along the middle axillary line using a Lap-Protector Mini (Hakko Co). The placement of the working port was tailored to the patient’s body shape, with special consideration given to female patients to avoid breast tissue and minimize postoperative discomfort.

Notably, the neoDRATS variant employs different configurations for the robotic arms.

Right-side configuration (Figure 1, *B* and *C*):

Arm 1: Left hand.

Arm 2: Right hand.

Arm 3: Camera.

Arm 4: Right hand (retraction).

Left-side configuration (Figure 2, *B* and *C*):

Arm 1: Left hand.

Arm 2: Camera.

Arm 3: Right hand (retraction).

Arm 4: Right hand.

These settings are the basic settings of the arms, and the retraction arm can be set to the left or right depending on the surgeon’s preference.

We consistently employed a 45 stapler with a curved tip (SureForm; Intuitive Surgical Inc) and opted for articulating staplers (Medtronic or Ethicon) when the assisting surgeon had adequate training. A robotic stapler is often used in neoDRATS because a well-trained assistant surgeon is not required. However, if a trained assistant surgeon cooperates, an articulating stapler can also be used. The use of a 60 stapler was avoided because of its limited internal angulation. Strategically placing another lower incision allows for better internal maneuverability of the staplers.

For the camera system, we consider either 30° or 0° acceptable; however, we prefer a 0° camera. Interference between the camera and the left and right arms can be avoided and the visible surgical field becomes wider with a 0° camera. Given the confined working space, careful placement of the camera and instruments within the incision is crucial to avoid collisions. By vertically adjusting the camera angle and arm placement, the left and right arms can be manipulated simultaneously, enabling unrestricted access throughout the thoracic cavity.

**Figure 3:**
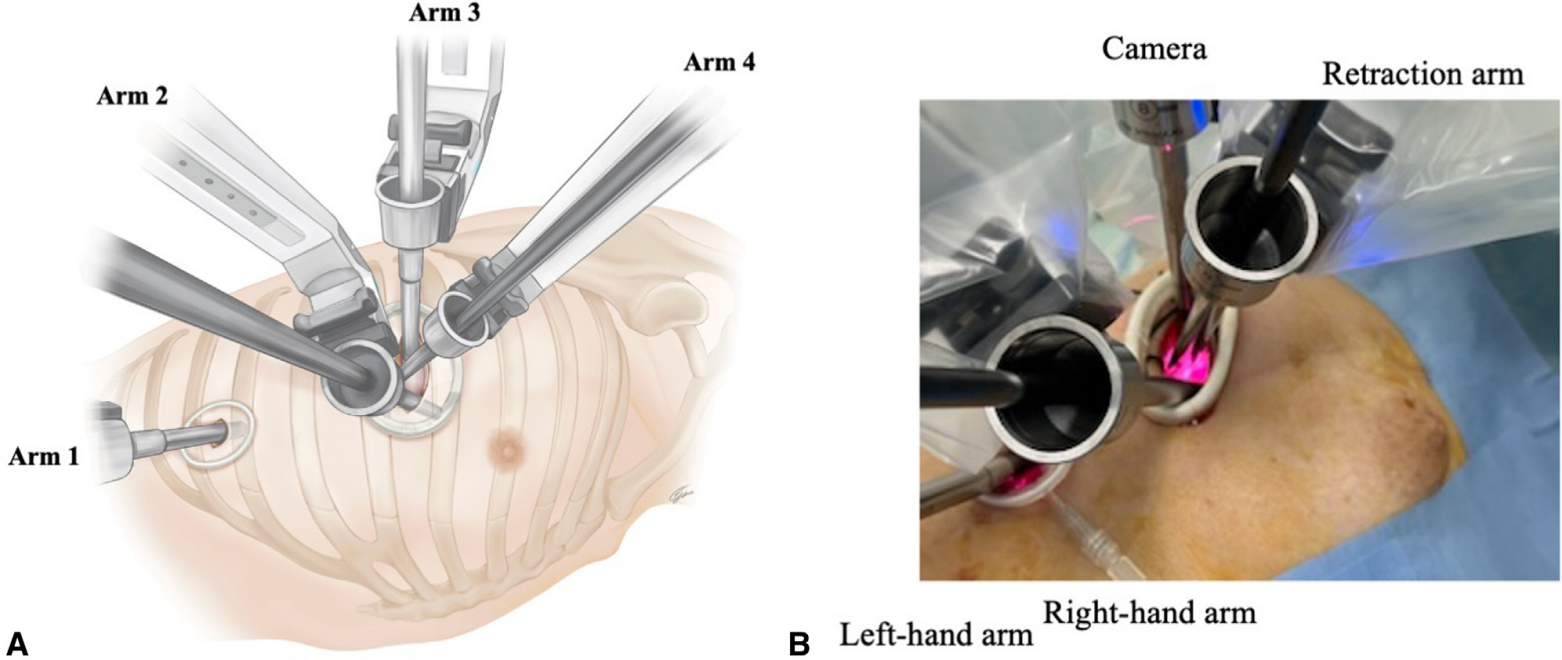
Right-side configuration. A, Illustration showing the port placement and setup of robotic arms on the right side. B, The arm placement in the working port is such that the camera is placed at the most dorsal side of the wound, the right arm in front of the wound, and the left arm inserted above the right arm to reduce interference in the thoracic cavity.

The specific robotic manipulations of the neoDRATS are as follows: The camera and the right and left arms are inserted through the working port, and the retraction arm is inserted through the second port. The main procedure is performed using the right and left arms inserted through the working port, and the surgical field is established using retraction-arm forceps. The arm placement in the working port is such that the camera is placed at the most dorsal side of the wound, the right arm in front of the wound, and the left arm inserted above the right arm to reduce interference in the thoracic cavity (Figure 3). When using a stapler, the retraction arm is changed, and it can be used in all areas of the thoracic cavity. Because the retraction arm may interfere with stapling, it is possible to remove the retraction arm and use the space in the thoracic cavity more efficiently. The assistant supports the operation by inserting curved suction and forceps through the working and secondary ports. If the assistant is skilled in UVATS, the surgical field can be improved.

Upon completion of the surgery, the assisting surgeon inserts a single chest drain toward the apex. The intraoperative neoDRATS approach for right upper lobectomy is demonstrated in Video 1.

## Discussion

Minimally invasive surgery is now the preferred method for treating lung cancer, with UVATS considered the least-invasive option for major lung resections. Conversely, the more technologically advanced RATS, which typically requires multiple incisions, is comparatively more invasive than single-incision UVATS.^4^ This paradox has led to the evolution of traditional RATS into URATS, which combines the UVATS techniques and is gaining international acceptance.^1^^,^^2^

However, URATS presents challenges, particularly with the need for surgical assistants to master UVATS techniques to prevent instrument collisions, emphasizing the assistant’s crucial role.^1^^,^^2^ This technique faces additional hurdles for patients with smaller thoracic cavities, where the limited space can hinder the insertion and angulation of staples, a situation often encountered in individuals with smaller body frames, such as those of Japanese heritage.

To address these issues, DRATS introduces an extra stapler port below the main incision, offering unrestricted stapler and forceps use within the thoracic cavity and doubling as an exit for a thoracic drain postsurgery.^3^ Furthermore, early findings suggest that DRATS may reduce postoperative pain and enhance outcomes compared with traditional 3-port RATS lobectomy.^5^ Despite its advantages, DRATS, like URATS, requires assistants skilled in UVATS because these procedures omit the use of the retraction arm found in conventional RATS.^5^

In the novel neoDRATS approach presented here, the 3 arms were inserted through the main incision, similar to URATS. However, the primary arms were operated from an additional port, reducing interference, and maintaining an optimal distance of 6 cm between the left and right arms, thereby ensuring smooth operability. However, depending on the side of the body and thoracic shape, the three arms interfered with each other at the working port. In such cases, the interference can be eliminated by limiting the number of arms in the working port to two and shifting to the conventional DRATS.

The limited adoption of URATS and UVATS in the United States is partly owing to the lack of skilled assistants (often physician assistants, nurses, or inexperienced residents). This limitation has led to a preference for solo surgery, which minimizes the assistant's role, over uniportal techniques.

NeoDRATS is designed to facilitate solo surgery even with less-experienced assistants, making it a potentially more viable option in areas where uniportal techniques are less common, such as the United States.

## Conclusions

Our experience highlights the substantial advantages of neoDRATS over URATS and DRATS, despite the challenges they entail. URATS poses difficulties, particularly in mastering UVATS techniques to prevent instrument collisions, and may present obstacles in patients with smaller thoracic cavities, such as those of Japanese descent. The surgery was performed after ensuring its safety. However, this study serves as an initial step toward refining neoDRATS for broader clinical integration. We believe that RATS will continue to evolve with further robotic innovations. Overall, we present neoDRATS as a practical, safe, feasible, efficient, and minimally invasive alternative to the conventional multiportal RATS.

## Conflict of Interest Statement

The authors reported no conflicts of interest.

The *Journal* policy requires editors and reviewers to disclose conflicts of interest and to decline handling or reviewing manuscripts for which they may have a conflict of interest. The editors and reviewers of this article have no conflicts of interest.

## Supplementary Data

Video 1 Intraoperative videos demonstrating the non-assistant help operation in dual-portal robotic-assisted thoracic surgery (neoDRATS) approach for a right upper lobectomy. Video available at: https://www.jtcvs.org/article/S2666-2507(24)00329-8/fulltext.

## References

[1] Gonzalez-Rivas D., Bosinceanu M., Manolache V. (2023). Uniportal fully robotic-assisted major pulmonary resections. Ann Cardiothorac Surg.

[2] Manolache V., Motas N., Bosinceanu M.L. (2023). Comparison of uniportal robotic-assisted thoracic surgery pulmonary anatomic resections with multiport robotic-assisted thoracic surgery: a multicenter study of the European experience. Ann Cardiothorac Surg.

[3] Watanabe H., Ebana H., Kanauchi N. (2023). Dual-portal robotic-assisted thoracic surgery (DRATS) as a reduced port RATS: early experiences in three institutions in Japan. J Thorac Dis.

[4] Cerfolio R.J., Bryant A.S., Skylizard L., Minnich D.J. (2011). Initial consecutive experience of completely portal robotic pulmonary resection with 4 arms. J Thorac Cardiovasc Surg.

[5] Han K.N., Lee J.H., Hong J.I., Kim H.K. (2022). Comparison of two-port and three-port approaches in robotic lobectomy for non–small cell lung cancer. World J Surg.

