# Supplementary figures and images for: Developing novel non-assistant help operation in dual-portal robotic-assisted thoracic surgery (neoDRATS)
Source: JTCVS Tech. 2024 Aug 5;27:146–50. doi: 10.1016/j.xjtc.2024.07.019 (PMC11518955; doi:10.1016/j.xjtc.2024.07.019)

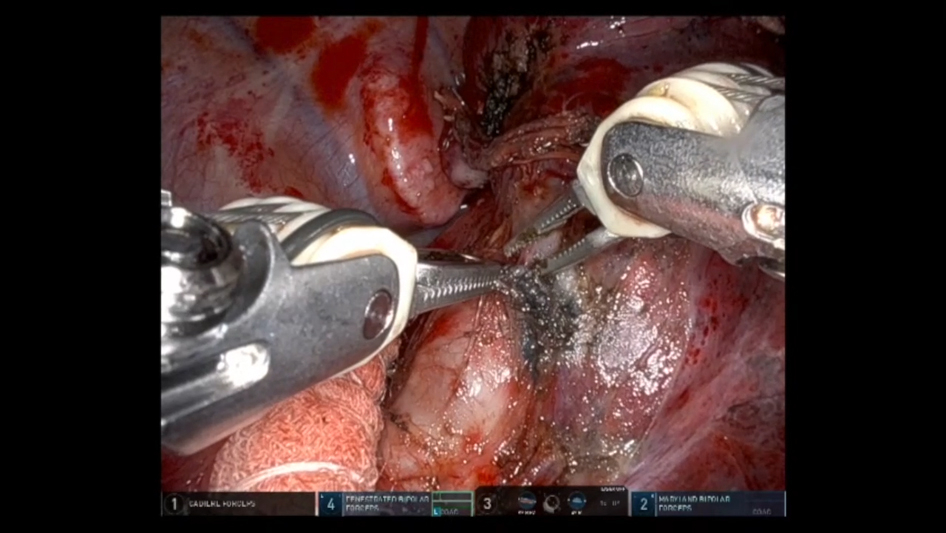

Supplement: Video 1 — Intraoperative videos demonstrating the non-assistant help operation in dual-portal robotic-assisted thoracic surgery (neoDRATS) approach for a right upper lobectomy. Video available at: https://www.jtcvs.org/article/S2666-2507(24)00329-8/fulltext. [file fx2.jpg]
